# Supplementary material for: Joint contact forces during barefoot, minimal and conventional shod running are highly individual
Source: Sci Rep. 2025 Jul 11;15:25022. doi: 10.1038/s41598-025-09174-w (PMC12254368; doi:10.1038/s41598-025-09174-w)
Supplement: Supplementary file 1 — Supplementary Information. [file 41598_2025_9174_MOESM1_ESM.pdf]

# Joint contact forces during barefoot, minimal and conventional shod running are highly individual.

Lena Kloock[1,+,*], Andrea Arensmann[1,+], Myriam de Graaf[1,2,3], Meike Gerlach[1], Kim Boström[1], and Heiko Wagner[1,2,3]

[1]Dept. of Movement Science, University of Münster, Horstmarer Landweg 62b, 48149 Münster, Germany
[2]Otto Creutzfeldt Center for Cognitive and Behavioral Neuroscience, University of Münster, Germany
[3]Center for Nonlinear Science (CeNoS), University of Münster, Correnssstraße 2, 48149, Münster, Germany
[*]lkloock@uni-muenster.de
[+]these authors contributed equally to this work

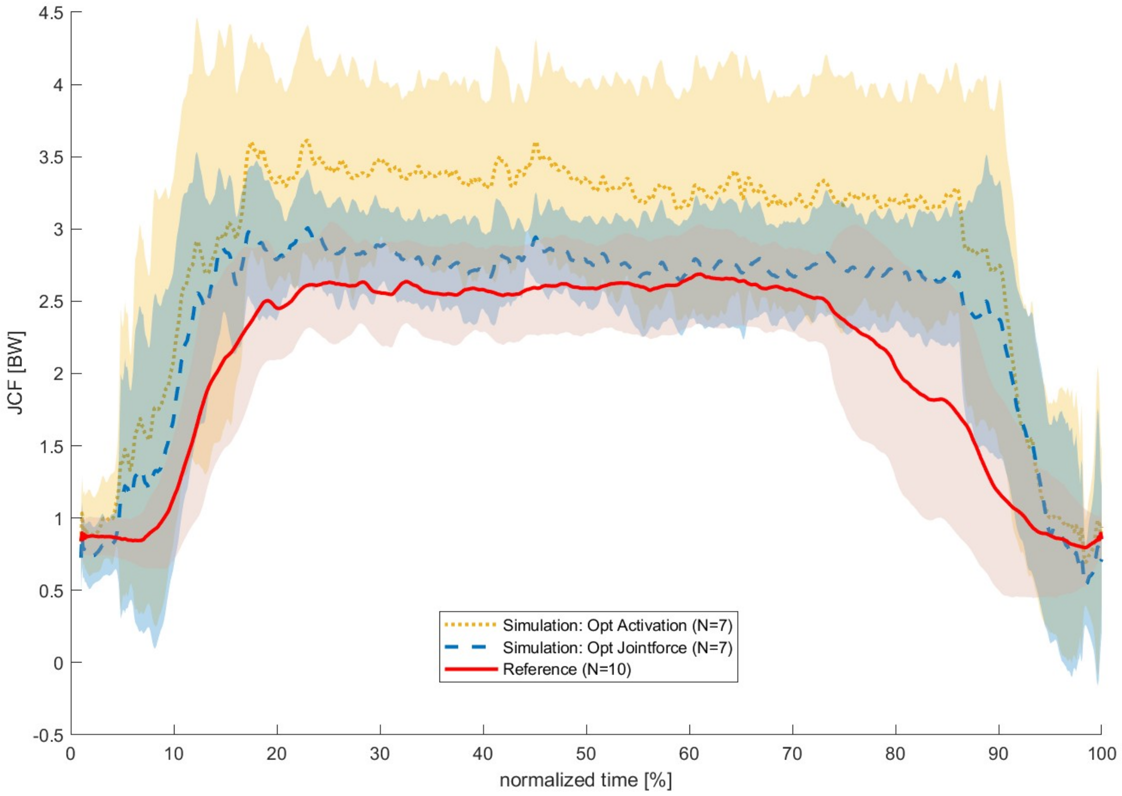

Figure S.1. Comparison of hip JCFs measured with an instrumented endoprosthesis (red) to rearranged simulated absolute joint contact forces (JCFs) of the hip with the Myonardo® (blue) during a one-legged stance as mean ± standard deviation.The movement data that was used for the calculation of the JCFs was collected with the same protocol as the reference data[1], but with younger, able bodies subjects without a previous hip replacement. In order to ensure a better comparison and to account for the impaired subjects of the validated data, the rearranged data was simulated using a muscle optimization to minimise the joint force, which was deemed appropriate for subjects with an impaired hip (opt: joint force). Additionally the rearranged data was simulated in the same manner that was used in the study, which is more appropriate for the subject group: a minimisation of the muscle activation[2]. All values were normalised to bodyweight and the time was normalised to fit all participants. Please note, that the reference data is from subjects aged 52 up until 68, while the subjects for the rearranged data were between 23 and 35, and it would thus be expected for them to have slightly higher hip contact forces[3]. The reference data was extracted from the database OrthoLoad[1] for 10 subjects (files, appended with 'one_leg_stance': H1L_060511_1_024, H2R_150811_1_024, H3L_141111_2_075, H4L_270_112_2_036, H5L_050412_1_037, H6R_201112_1_036, H7R_191112_1_038, H8L_240413_1_036, H9L_301013_1_029, H10R_300114_1_032).

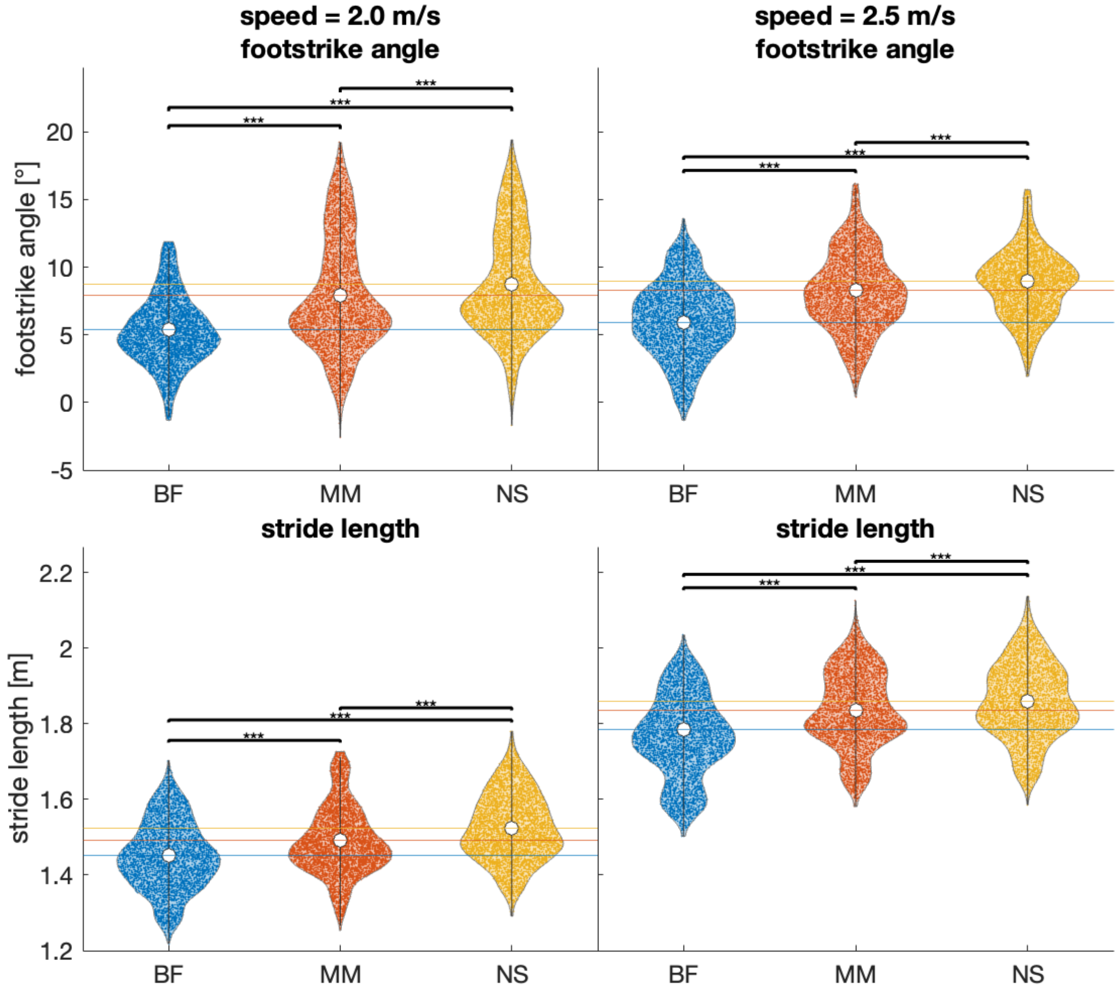

Figure S.2. Footstrike angle (top) and stride length (bottom) of all steps of all participants per footwear condition (BF = barefoot, MM = minimal shoes, NS = normal shoes) in both speeds (2.0 m/s left, 2.5 m/s right). Each data point represents one stride. Additionally, the median values are indicated by a white dot, and the interquartile range as a grey bar. Significance between conditions is indicated using asterisks: $^{*}p < 0.05$, $^{**}p < 0.01$, $^{***}p < 0.001$

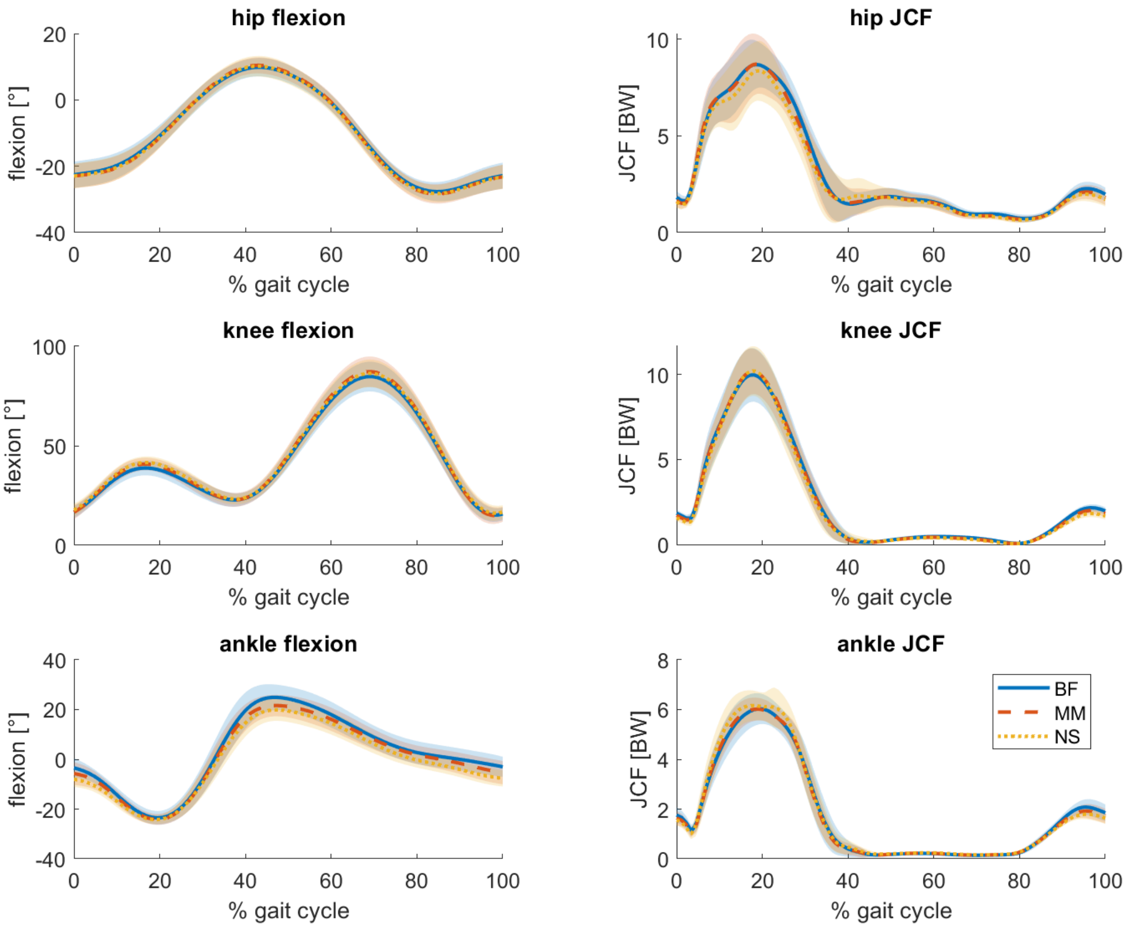

Figure S.3. Joint flexion angles (left) and joint contact forces (right) per footwear condition as a function of the stride cycle during running at 2.5 m/s. Values are averaged over all steps of all subjects (n=16) for the hip (top), knee (middle) and ankle (bottom) per condition. Barefoot running (BF) is indicated with a solid blue line, minimal shoes (MM) with a dashed orange, and normal shoes (NS) with a dotted yellow line. An angle of zero indicates the joint angles in the anatomical position. Positive angles indicate increased extension at the hip, increased flexion at the knee and increased plantarflexion at the ankle. Shaded areas indicate the standard deviation around the mean.

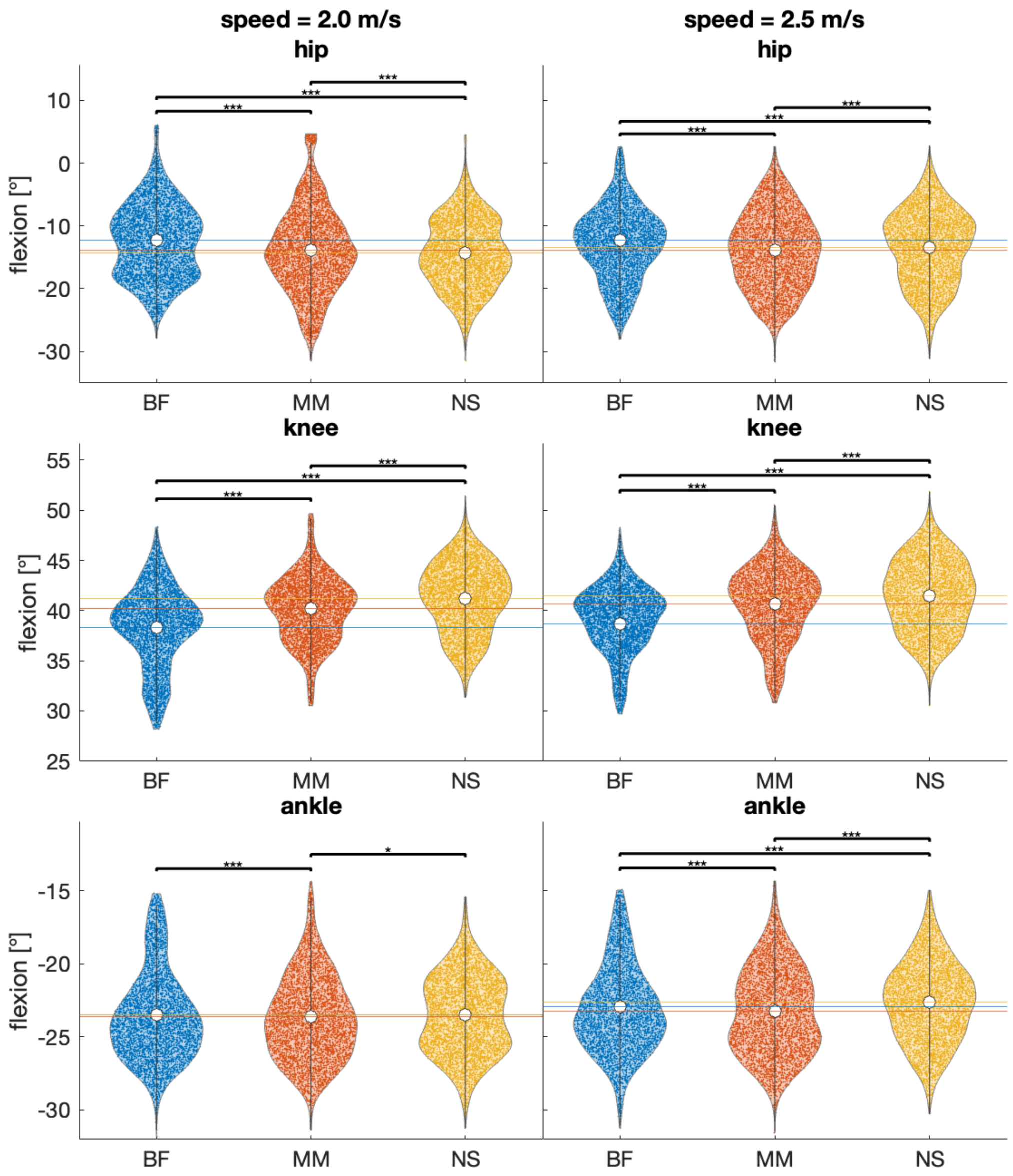

Figure S.4. Flexion angles at peak joint contact force (JCF) of all steps of all participants per footwear condition (BF = barefoot, MM = minimal shoes, NS = normal shoes) in both speeds (2.0 m/s left, 2.5 m/s right) and for all joints (hip: top, knee: middle, ankle: bottom). Each data point represents one stride. Additionally, the median values are indicated by a white dot, and the interquartile range as a grey bar. Significance between conditions is indicated using asterisks: $^{*}p < 0.05$, $^{**}p < 0.01$, $^{***}p < 0.001$

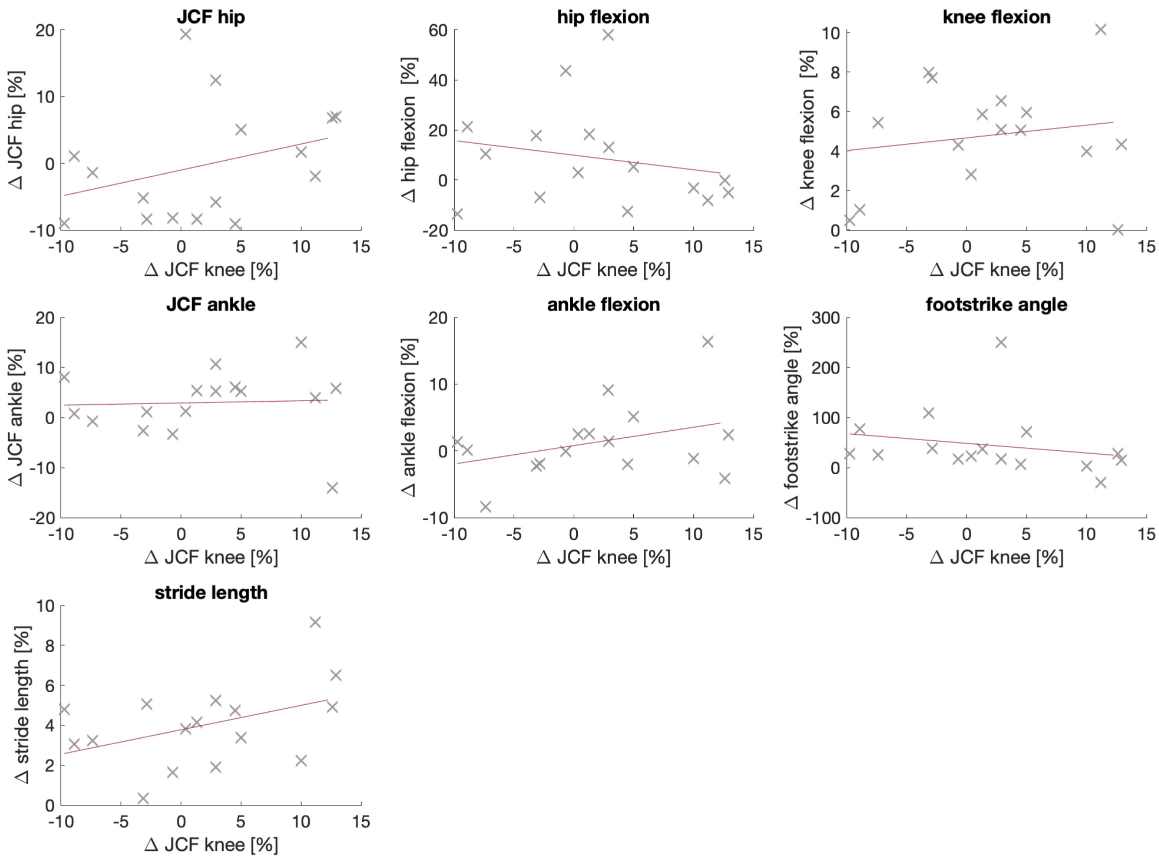

Figure S.5. Linear regression between the percent change in joint contact force (JCFs) at the knee and the percent change in all other variables from BF to MM at 2.5 m/s. Each cross represents the response of one individual. Correlation analysis was performed for these variables and yielded no significant correlations

References

1. Bergmann, G. & Damm, P. (eds.). Julius Wolff Institute, Berlin Institute of Health at Charité Universitätsmedizin Berlin (2008). "OrthoLoad". Retrieved Feb. 1, 2009 from http://www.OrthoLoad.com.
2. Zargham, A., Afschrift, M., De Schutter, J., Jonkers, I. & De Groote, F. Inverse dynamic estimates of muscle recruitment and joint contact forces are more realistic when minimizing muscle activity rather than metabolic energy or contact forces. *Gait & Posture* **74**, 223–230, DOI: 10.1016/j.gaitpost.2019.08.019 (2019).
3. Williams, D. S. B. & Powell, D. W. Smaller Compressive and Anteroposterior Joint Reaction Forces in Older Compared to Young Runners: 482 Board #303 May 31 9 30 AM - 11 00 AM. *Medicine & Sci. Sports & Exerc.* **49**, 133, DOI: 10.1249/01.mss.0000517192.61130.36 (2017).
